# Cardiovascular Information and Health Engineering Medicine

**DOI:** 10.34133/research.0956

**Published:** 2025-10-17

**Authors:** Xue Bao, Ziyu Zhang, Yuhe Shen, Peng Tang, Guangxiang Si, Lina Kang, Biao Xu, Ning Gu

**Affiliations:** ^1^ Department of Cardiology, Cardiovascular Disease Center, Jiangsu Key Laboratory for Cardiovascular Information and Health Engineering Medicine, Nanjing Drum Tower Hospital, Medical School, Nanjing University, Nanjing 210093, P. R. China.; ^2^ Nanjing Research Center for Biomedical Electron Microscopy (NRC-BEM), Nanjing Drum Tower Hospital, Medical School, Nanjing University, Nanjing 210093, P. R. China.

## Abstract

Cardiovascular diseases (CVDs) persistently impose a substantial global health burden, highlighting the necessity for innovative approaches to their prevention, diagnosis, and treatment. This paper accentuates the close correlation between vascular health and CVDs. To address unmet clinical demands for the early and efficient management of CVDs, we emphasize the potential of engineering medicine and propose the concept of cardiovascular information and health engineering medicine (CVIHEM), delineating its objectives and framework for collecting, analyzing, and translating multidimensional vascular data into practical strategies. By systematically and efficiently acquiring and translating vascular information through multidisciplinary collaboration, CVIHEM enhances our understanding of the vascular network and provides innovative strategies for promoting vascular health. This approach encompasses a range of applications, such as artificial intelligence-driven decision-making, targeted therapy, and vascular health management, ultimately improving vascular health and clinical outcomes.

## Introduction

### Burden of cardiovascular diseases

Cardiovascular diseases (CVDs) are the leading cause of mortality and morbidity worldwide. According to the Global Burden of Disease study, a total of 614.51 million people had CVDs in 2021, which represents an increase of 33.64% from 2010. Additionally, there were 19.91 million deaths attributed to CVDs in 2021, representing an increase of 21.56% compared to 2010 [1]. CVDs also pose a substantial economic burden. The total cost of hospitalizations for CVDs in China amounts to 270.9 billion RMB, while in the United States, direct expenditures on CVDs are estimated at $254.3 billion [2,3]. With an aging population and ongoing risk factors, the burden of CVDs is expected to rise, with a projection of a 90.0% increase in prevalence, a 73.4% increase in crude mortality, and a 54.7% increase in crude disability-adjusted life years from 2025 to 2050 [4].

### Unmet clinical demands in the management of CVDs

Despite notable progress in medical research and management of CVDs, considerable challenges remain. (a) Insufficiently sensitive and accurate prediction and early-stage diagnosis: The progression of CVDs is usually slow, spanning several years or even decades. They can be asymptomatic in the early years, but late-stage phenotypes like acute coronary syndrome or heart failure can be sudden and fatal. Traditional diagnostic procedures, such as blood pressure measurement, lipid analysis, electrocardiograms, and echocardiography, often lack sufficient sensitivity and specificity, resulting in late diagnoses with more limited treatment options and less favorable prognosis. While being crucial for CVD diagnosis, cardiovascular imaging has technical constraints like contrast agent risks [5], motion artifacts [6], and relatively low-resolution and inadequate ability to capture valuable biochemical and mechanical information (such as plaque vulnerability [7]) or correlate it with clinical phenotypes. Developing better early indicators to address these challenges is essential to advance the inflection point of CVDs. (b) Targeted treatment and theranostics: Despite the identification of numerous molecular targets for CVDs in experiments, converting these findings into effective therapeutic strategies is difficult. Moreover, theranostics, which integrates treatment and diagnosis to promptly identify and address abnormalities before they deteriorate, is vigorously pursued in CVD treatment. The emerging nanotechnology offers potential but still faces many challenges [8,9]. Urgent further research is needed to explore potential toxicity, enhance nanoparticle design, increase production capacity, and investigate diverse synergistic imaging and therapeutic methods.

## Vascular Aging and CVDs

To address the urgent need for alleviating the CVD burden, a shift in healthcare focus from late-stage treatment and remediation to early prevention and intervention strategies is imperative. Understanding the underlying mechanisms of CVD development is of utmost significance. In this regard, vascular aging emerges as a crucial factor. As we delve deeper into this subject, it becomes evident that uncovering the intricate connection between vascular aging and CVDs is crucial to achieving more effective prevention, early diagnosis, and treatment strategies.

The vascular network is a versatile system comprising arteries, capillaries, veins, and lymphatic vessels that functions under the driving force of the heart, ensuring effective circulation [10]. This system is primarily recognized for its roles in tissue oxygenation, metabolism, and immune surveillance [10]. The endothelium, which lines the blood vessels, regulates vascular tone, angiogenesis, and hemostasis, and maintains an antioxidant, anti-inflammatory, and antithrombotic environment [11]. Notably, the cardiovascular, immune, and nervous systems are highly integrated [12,13]. Together, they play an important role in regulating development and physiological functions. Disruptions in their balance can lead to various pathological conditions, such as electrical instability [14], atherosclerosis [15], and cognitive decline [16]. Additionally, the vasculature influences the behavior of neighboring cells by delivering local, often organ-specific, molecular signals [17] while also facilitating interactions among distant organs by enabling complex biological transmission [18]. Therefore, the vascular network plays a pivotal role in maintaining overall homeostasis.

Over 3 centuries ago, Thomas Sydenham (1624–1689), often called the “English Hippocrates”, stated, “A man is as old as his arteries”. This quote implies that the condition of blood vessels, rather than chronological age, profoundly affects the overall health and is intimately involved in the pathology of age-related alterations. Circulating progeronic and antigeronic factors from various organs and systems (including the brain, endocrine system, immune system, and adipose tissue) collectively orchestrate aging processes within large vessels and microcirculation, accompanied by fundamental cellular and molecular mechanisms in the vascular system. The resulting functional dysregulation associated with vascular aging contributes to a range of age-related pathologies, with CVDs serving as a key example [19]. Recognizing the systematic impact of vascular dysregulation on age-related diseases, the concept of “panvascular diseases” has emerged as a perspective to investigate CVDs based on their shared etiologies and characteristics [20,21]. Unlike chronological age, vascular age can be influenced by various factors, including lifestyle behaviors (smoking, physical activity, diet, and weight) and health markers (cholesterol, blood pressure, and glucose control) [3]. Numerous efforts have been made to understand vascular age and identify its proxies [22]. One classical example is the estimation of vascular aging using CVD algorithms calculated from traditional risk factors in the Framingham Heart Study [23]. Recently, the Kailuan Study, involving 20,917 middle-aged Chinese participants, calculated vascular age using traditional cardiovascular risk factors and pulsed wave velocity. The results indicated that for each 1-year increase in the difference between chronological age and vascular age, there was a 26% reduction in the risk of cardiovascular events [24]. In addition, structural and functional arterial properties, such as plaque burden, intima–media thickness, coronary artery calcium, and carotid–femoral pulse wave velocity (PWV), have also been used as the proxies of vascular aging [22]. However, it is important to note that the information carried by vascular aging goes far beyond traditional risk factors and other overt parameters.

## Engineering Medicine: A Powerful Tool to Acquire and Translate Vascular Information

**Fig. 1.**
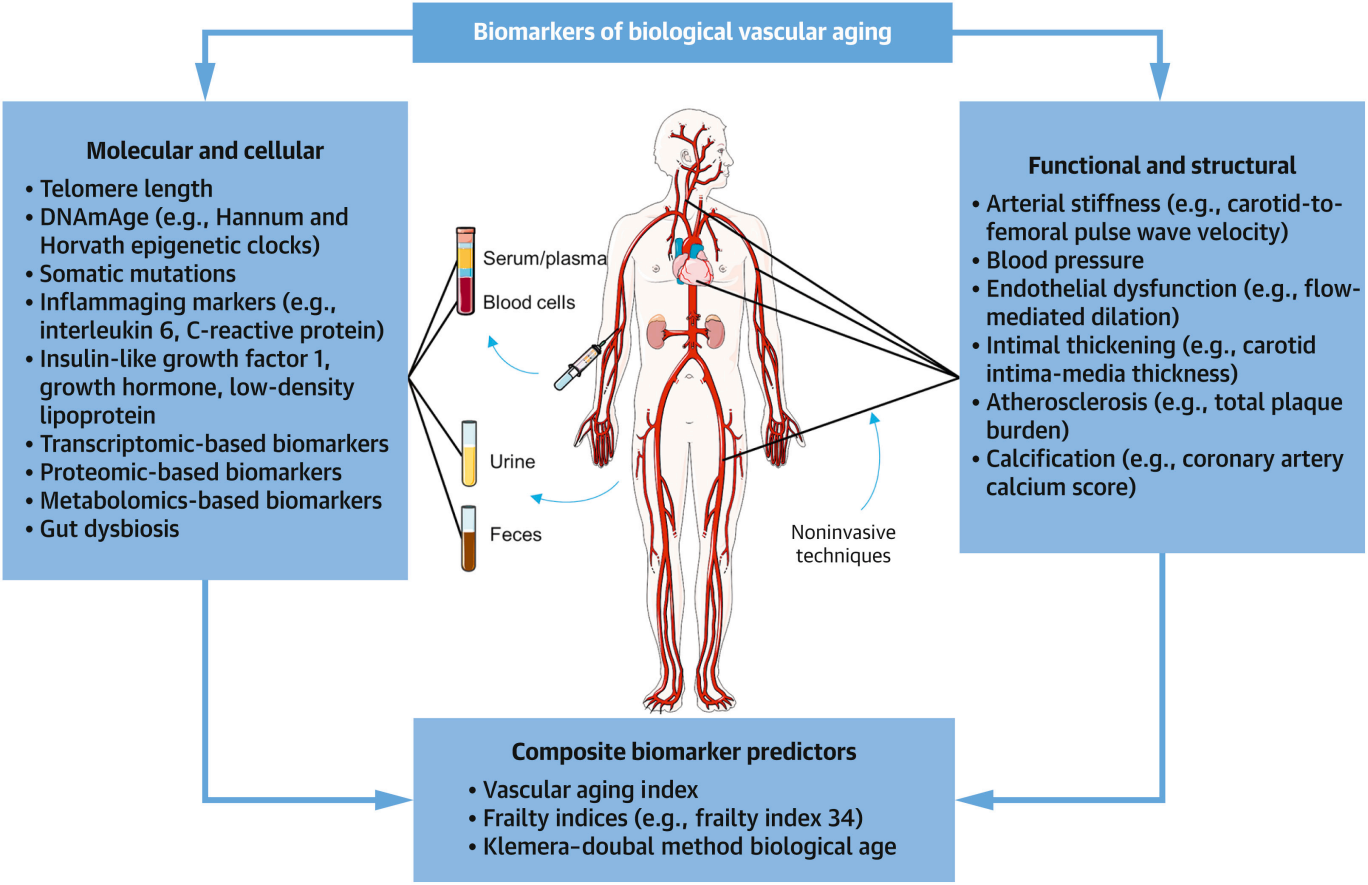
Biomarkers of biological vascular aging. The biological age of the vasculature can be estimated using molecular and cellular biomarkers (in blood, urine, or feces), functional and structural vascular parameters (measured by noninvasive techniques), or a combination of both. DNAmAge, DNA methylation age. Reprinted with permission from Elsevier [28].

**Fig. 2.**
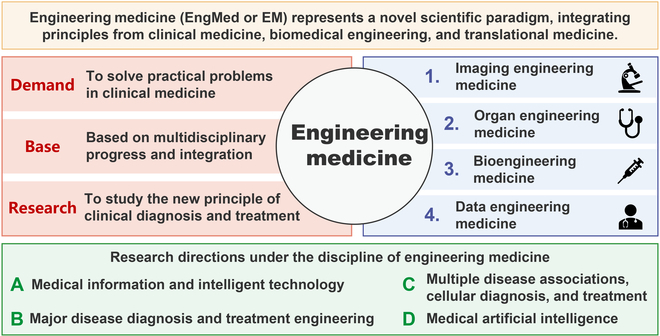
Main tasks of engineering medicine. Adapted from [30].

Numerous complex metabolic and hemodynamic changes disrupt cardiovascular homeostasis with vascular aging, leading to impaired cardiovascular health. These dysregulations can manifest systematically and are accompanied by intricate molecular and cellular processes [25–27], providing a wealth of potential biomarkers for the early prediction of CVDs. A wide range of biomarkers for vascular aging has been proposed and can be categorized into functional, structural, and humoral dimensions [28,29] (Fig. 1). However, current clinical methods often fail to capture adequate vascular information or translate it efficiently into improved diagnoses and treatments for CVDs. In this context, the pioneering scientific paradigm of engineering medicine (EngMed) [30], originally conceptualized by our team in early 2025, offers great potential to advance vascular data interpretation and management. The review paper [30] systematically explored potential applications of EngMed, covering imaging, organ modeling, bioengineering, and digital health fields. Through interdisciplinary integration, EngMed tackles clinical challenges with innovative biomedical engineering tools and methodologies, facilitating the continuous improvement of disease diagnosis and treatment modalities (Fig. 2).

## Cardiovascular Information and Health Engineering Medicine

The application of EngMed [30] in the field of vascular health engenders a novel scientific framework called cardiovascular information and health engineering medicine (CVIHEM). CVIHEM is an interdisciplinary field dedicated to systematically acquiring, analyzing, and translating multidimensional vascular data (e.g., imaging, sensing, and functional) into actionable clinical strategies for vascular disease management. Its theoretical boundaries are defined by 3 core pillars: the integration of medical knowledge (vascular pathophysiology), engineering methods (nanodevices, computational modeling), and health informatics (data integration); a closed-loop information translation mechanism where engineering analysis [artificial intelligence (AI) modeling, simulation] converts raw vascular data into targeted therapies, tissue engineering solutions, or health management protocols, with real-world validation iteratively refining the data pipeline; and a distinct focus on vascular health as opposed to broader cardiovascular engineering, emphasizing efficient, interdisciplinary convergence to advance early diagnosis, treatment innovation, and holistic vascular health promotion.

**Fig. 3.**
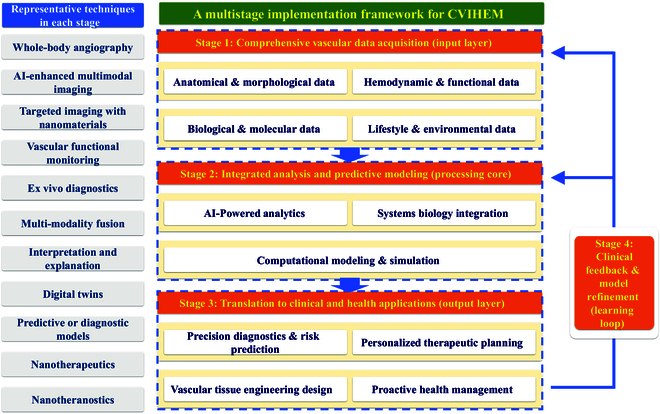
Framework of cardiovascular information and health engineering medicine. AI, artificial intelligence.

Figure 3 outlines a 4-stage workflow for the CVIHEM implementation pathway, with each stage enabled by EngMed techniques. Stage 1 serves as the input layer, systematically collecting high-dimensional longitudinal data that comprehensively characterize an individual’s vascular and related systems through advanced imaging, vascular functional monitoring, ex vivo diagnostics, and related modalities. Stage 2 functions as the processing core, translating raw multidimensional data from stage 1 into actionable insights and predictive models primarily using AI techniques. Stage 3 operates as the output layer, applying insights and models derived from stage 2 to address unmet clinical needs, such as predicting cardiovascular risk, simulating stent hemodynamics, designing biomechanically optimized vascular grafts, generating personalized prevention strategies, and developing targeted therapies. In stage 4, real-world outcomes from stage 3 interventions are captured and fed back into the system to iteratively retrain and refine strategies and models in preceding stages, thereby establishing the CVIHEM framework as a cyclical, learning-oriented system.

### Merging CVIHEM technologies: Opportunities and ongoing challenges

Previous reviews have already provided detailed discussions in multiple related technologies, such as vascular imaging [31], medical AI [32], and nanotherapeutics [33], within their respective technical domains. Instead of repeating these technical details, the following section of this paper focuses on key innovations that support each stage of the CVIHEM framework. Table 1 provides a structured overview of representative technologies at each stage, delineating their advantages, challenges, and solutions, emphasizing how integrated CVIHEM framework drives translational outcomes from data acquisition to clinical application.

**Table 1.** Advantages, challenges, and future solutions of representative technologies at each stage of the CVIHEM framework

Technical field	Core advantages	Major challenges	Root causes	Future solutions
Stage 1: Comprehensive vascular data acquisition (input layer)				
Ferumoxytol-enhanced whole-body angiography ^a^	High-efficiency, low-dose imaging Extended imaging window No nephrotoxicity Molecular imaging capability	Susceptibility to imaging artifacts Subvoxel vessel detection limitations Arteriovenous synchronization challenges	Inadequate artifact reduction algorithms MRI resolution constraints Arteriovenous synchronization in imaging	Technical advancements (arteriovenous separation algorithms, dynamic SWI fusion) Advanced postprocessing (self-calibrating/motion-compensated reconstruction)
Multimodal imaging and AI ^b^	Enhanced CVD prediction/diagnosis	Technical complexity Modality heterogeneity Partial modality missing	Vascular topological complexity High data annotation costs Limited algorithm robustness Medicine–engineering collaboration gaps	Standardized multi-modal datasets Advanced algorithms (GANs/foundation models) Cross-disciplinary validation consortia
Targeted imaging with nanomaterials ^a^	Tunable physicochemical properties for contrast enhancement Multimodal imaging integration	-	-	-
Wearable vascular monitoring	Continuous noninvasive biosignal monitoring Multimodal sensing integration Real-time ML-driven analytics	Limited clinical reliability Frequent calibration needs Motion artifacts	Sensor material limitations Complex physiological signal noise Anatomical/physiological variability	Advanced flexible biosensor materials Deep learning noise reduction Personalized calibration frameworks
Nanomaterial-based ex vivo diagnostics	Enhanced biomarker sensitivity Cost-effective rapid screening Multiplexed biomarker profiling	Background noise in complex matrices Unvalidated CVD biomarkers Clinical integration barriers	Biological sample complexity Incomplete biomarker-pathway understanding Analytical technique limitations	Surface functionalization for signal boost AI-driven biomarker discovery Standardized diagnostic–therapeutic interfaces
Stage 2: Integrated analysis and predictive modeling (processing core)				
Unified fusion ^b^	Topology-preserving multi-modal alignment Low-dose/contrast robustness Uncertainty-aware completion	Hallucinated structures	Physics-agnostic priors Parameter non-identifiability	Physics-informed priors Multi-fidelity curricula
Interpretable modeling ^b^	Concept-aligned reasoning Transparent and auditable Physiology-aware constraints	Weak physiologic/morphologic grounding	Post hoc detachment from physics Single-modality heuristics	Intrinsic concept models Multi-modal OOD and consistency checks
Digital twins ^b^	Patient-specific hemodynamics Physics-plausible surrogates progression-aware planning	Real-time latency	Scale mismatch and nonconvexity High-dimensional PDE/ODEs and edge limits	Stable joint training with uncertainty Fast-slow surrogates and online assimilation
Stage 3: Translation to clinical and health applications (output layer)				
AI-driven decision strategies ^b^	Precision diagnostics and personalized therapy planning Closed-loop across diagnosis–treatment–follow-up	Complex cross-pathway integration	limited interpretability Missing interoperability and governance Incomplete real-world feedback and retraining pipelines	-
Nanotherapeutics ^a^	Targeted delivery Sustained release Enhanced functional biomimicry	-	-	-
Nanotheranostics ^a^	Integrated diagnosis and therapy Real-time efficacy monitoring	Imaging-therapy mismatch Complex manufacturing	Technical integration	Systematic studies on the therapeutic and imaging performances and PK/PD Novelty pursuit vs. practical optimization balance
Stage 4: Clinical feedback and model refinement (learning loop)				
Feedback optimization	Cross-institutional data interoperability Decision process optimization Closed-loop decision support	System interoperability challenges Compliance monitoring lag	Fragmented healthcare information standards Lack of post-deployment monitoring and retraining processes	Modular API architecture AI-driven compliance monitoring and evaluation
Common issues
Nanomaterials	-	Low targeting efficiency Toxicity risks Synthesis complexity Scalability issues	Physiological barriers (vascular permeability, MPS uptake) Material instability Bio-interaction uncertainty Biological complexity High R&D costs Lack of standardization Regulatory hurdles	Targeting optimization (stealth coatings and high-specificity ligands) AI-driven design Toxicity mitigation (heavy metal-free or clearable) Safety protocols Synthesis standardization Encourage simplicity for practical implementation Early regulatory engagement
AI models	-	Cross-device/cross-population/cross center performance decay Cross-site and deployment instability Prolonged clinical validation Unstable hybrid training	Center/protocol/vendor data heterogeneity Heterogeneous deploy pipeline Pathological topological overfitting	Federated learning validation and drift monitoring API interoperability Enhanced interpretability and causal reasoning
CVIHEM paradigm	Multidimensional vascular data “acquire–analyze–translate–feedback” closed loop, enabling precision-focused and prevention-oriented vascular health management	Challenges of all related technologies mentioned above	Root causes of all related technologies mentioned above	Establish engineering–medical collaboration platform Develop CVIHEM validation standards Future solutions for all related technologies mentioned above

AI, artificial intelligence; API, application programming interface; CVD, cardiovascular disease; CVIHEM, cardiovascular information and health engineering medicine; GANs, generative adversarial networks; MRI, magnetic resonance imaging; MPS, mononuclear phagocyte system; OOD, out-of-distribution; PDE/ODE, partial differential equations/ordinary differential equations; PK/PD, pharmacokinetic/pharmacodynamic; R&D, research and development

^a^ Common issues of nanomaterials are detailed in the Common issues section of this table.

^b^ Common issues of AI models are detailed in the Common issues section of this table.

#### Stage 1: Comprehensive vascular data acquisition (input layer)

Stage 1 is dedicated to capturing high-dimensional vascular data across multiple spatial and temporal scales. The following outlines several representative technologies employed in vascular data acquisition.

##### Whole-body angiography

**Fig. 4.**
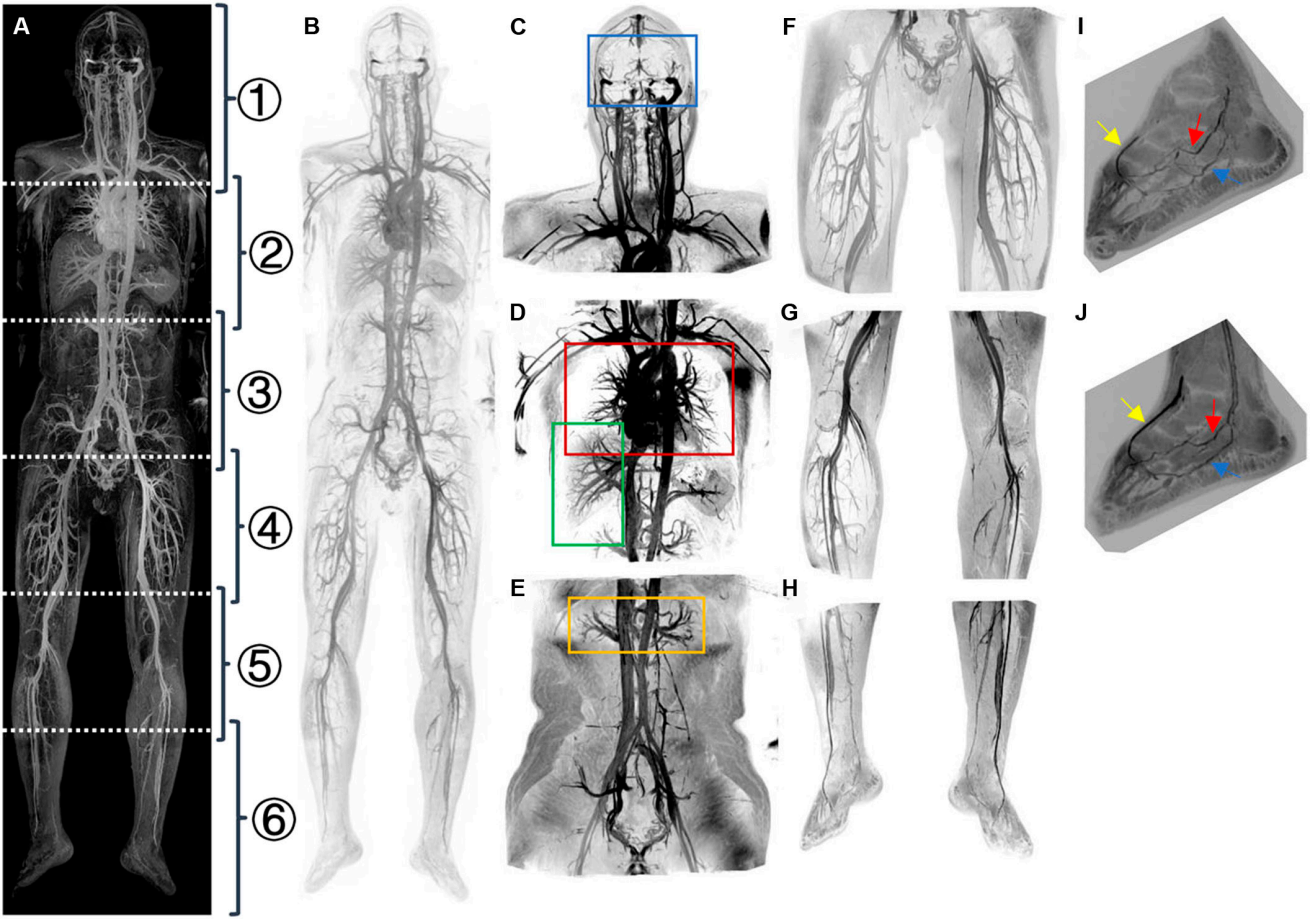
Representative images of ferumoxytol-enhanced whole-body magnetic resonance angiography with 3.0 mg/kg ferumoxytol at 0.1 h post-administration. (A) Maximum intensity projection (MIP) generated from 6 stitched raw image segments. (B) Inverted grayscale of (A) to emphasize vessel enhancement. (C to H) MIPs of the head and neck, thorax, abdomen, and pelvis, and upper to distal portions of the lower extremity. Small vessels are highlighted in various regions (blue, red, green, and yellow rectangles). (I and J) Magnified views of right and left feet, revealing fine peripheral vessels like the medial plantar artery (red arrows), lateral plantar artery (blue arrows), and dorsalis pedis artery (yellow arrows). Reprinted with permission from [37].

Whole-body angiography provides a comprehensive view of vascular health and changes, directly supporting stage 1’s requirement for whole-network vascular mapping in CVIHEM framework. Advancements in scanner hardware, spatial and temporal resolution, table length, and motion scanning have markedly enhanced the clinical feasibility of whole-body magnetic resonance angiography (WB-MRA). This modality offers superior visualization of deep vascular structures compared to ultrasound while avoiding ionizing radiation exposure associated with computed tomography (CT) [34]. Contrast agents have greatly improved imaging quality and have become an essential component of clinical imaging protocols [35]. Among clinical approved intravenous magnetic resonance imaging (MRI) contrast agents (gadolinium-, iron-, and manganese-based), superparamagnetic iron oxide distinguishes itself in vascular imaging due to its superior safety profile and remarkable imaging efficacy [36]. Notably, its prolonged intravascular half-life holds substantial potential for WB-MRA applications [8,34]. Ferumoxytol, while Food and Drug Administration-approved for intravenous iron replacement therapy, has emerged as one of the most widely utilized iron-based agent for off-label MRI contrast enhancement. Emerging evidence supports its utility for WB-MRA [37–39]. A recent pilot study [37] indicates that ferumoxytol-enhanced WB-MRA can achieve prolonged vascular enhancement for at least 48 h at lower dosages, enabling sustained enhancement of the vasculature throughout the entire body (Fig. 4). Despite its advantages (Table 1), ferumoxytol-enhanced WB-MRA faces challenges such as artifact susceptibility (e.g., motion artifacts from cardiorespiratory dynamics and metal artifacts from implants), arteriovenous differentiation on steady-state images [40], and detection of subvoxel vessels, driving future advancements toward AI-driven algorithms and computational modeling for artifact reduction, arteriovenous separation [41], and subvoxel vessel detection [42].

##### AI-enhanced multimodal imaging

Traditionally, cardiovascular imaging evaluations have been performed independently, each with its inherent limitations [43,44]. Multimodality imaging enhances diagnosis and management of CVDs through synergistic data integration from complementary modalities [45,46]. It also substantially reduces the time commitment per examination, thereby optimizing diagnostic throughput and clinical decision-making efficiency [47,48]. This approach importantly facilitates a comprehensive characterization of vascular signatures in stage 1 of the CVIHEM framework. Registration, segmentation, and fusion present core technical challenges in multimodal imaging, where AI demonstrates transformative potential through [49]. For instance, advancements in cardiovascular image processing and analysis have driven the development of automated segmentation algorithms [50,51], supported by publicly available cardiovascular datasets [51,52]. Multimodal imaging along with AI analysis has become increasingly prevalent as summarized in Table S1 [47,48,50,53–56]. However, key challenges remain, including partial modality missing in real-world scenarios, data heterogeneity, and cross-modal fusion difficulties arising from disparities in spatial resolution, contrast mechanisms, and imaging principles (Table 1) [49]. The complexity of vascular networks also poses technical challenges, such as vascular topological complexity especially under pathological conditions (e.g., stenosis/aneurysms) and segmentation discontinuities caused by low contrast-to-noise ratios (particularly in small vessels). Critically, AI models trained on specific datasets show limited generalization, with performance drops across modalities, demographics, or imaging devices, highlighting the need for domain adaptation techniques. Future efforts should focus on standardizing multi-modal datasets, using generative AI (e.g., generative adversarial networks) for synthetic modality completion, and cross-disciplinary collaborations to validate robust solutions, ultimately enhancing cardiovascular risk prediction, pathology segmentation, and anatomical labeling.

##### Targeted imaging with nanomaterials

Nanotechnology helps in balancing imaging depth and specificity in stage 1 of the CVIHEM framework via its ability to target specific cell subsets or tissues of interest, facilitating the imaging of distinct vascular changes, such as early or vulnerable plaques or vascular calcification [57,58]. Preclinical CVD nanoimaging uses magnetic, nuclear, acoustic, and optical nanomaterials and combinations thereof to generate contrast tailored to specific imaging modalities, such as iron oxide nanoparticles (IONPs) for MRI and magnetic particle imaging (MPI) and gold nanomaterials for CT [57]. Strategies have been developed to design nanoparticles that target various molecular characteristics [8,57], such as apoptosis, atherosclerosis-related lipids and extracellular matrix proteins, endothelial activation, neoangiogenesis, calcium deposition, and notably immune-related phenomena, which play a critical role in vascular pathologies [59,60]. Photoacoustic imaging using single-walled carbon nanotubes exploits optical absorption for sensitive monocyte trafficking detection in carotids [61]. Nuclear approaches like ^19^F-MRI [62] and ^89^Zr-positron emission tomography (PET) [63] track myeloid cell dynamics from splenic reservoirs to plaques. Novel approaches such as “smart nanomaterials” activated by triggers or multimodal strategies are also being tested [57]. Magnetic approaches stand out for their noninvasiveness and high resolution. IONP-based agents target macrophage phagocytosis or apoptotic markers to detect plaque vulnerability via MRI [36]. Emerging MPI enables real-time multicolor quantitative tracking with minimal doses and avoiding ionizing radiation [64]. However, common challenges of nanomaterials remain in nanomaterial-targeted imaging [57,65] (Table 1), including low delivery efficiency, potential toxicity, and limited synthesis scalability. Emerging strategies like AI-guided design, biodegradable alternatives, and standardized synthesis protocols aim to enhance precision and safety. Concurrently, early regulatory engagement and cross-sector collaboration will be critical to streamline clinical translation and unlock scalable, cost-effective solutions for cardiovascular applications.

##### Vascular functional monitoring

Vascular function assessment is pivotal for CVD screening and management. It serves as an essential modality for characterizing the dynamic spatiotemporal patterns of vascular functionality required in stage 1 of the CVIHEM framework. A variety of vascular function assessments are currently used in clinical practice. Invasive methods involving intra-arterial infusion of vasoactive substances and direct assessment of vascular response via catheterization-based methods are not widely applicable due to procedural risks and costs [66]. Noninvasive techniques include physiological tests such as flow-mediated vasodilation (FMD), reactive hyperemia index, strain-gauge pulse plethysmographs, PWV, augmentation index, intima media thickness, and chemical biomarkers as supplementary tests [67]. PWV is commonly used to assess arterial stiffness [67,68], while FMD is widely used to measure endothelial function [66,67,69], although it is technically challenging and requires extensive training and standardization [69]. Newer techniques, such as finger plethysmography and the retinal flicker test, also show potential for future application [69]. Moreover, emerging CVIHEM solutions overcome the limitation of traditional techniques, such as spatiotemporal resolution constraints (single-timepoint measurements, hospital-centric workflows) and invasiveness by leverage advancements in flexible electronics, Bluetooth low energy, biocompatible materials, and machine learning algorithms. These innovations enable the development of wearable biosensors capable of continuous, ambulatory monitoring of pulse waves, blood volumetric changes, ultrasonic signals, electrocardiogram, and blood pressure (Table 1) [70–72]. However, achieving clinical-grade accuracy requires overcoming technical challenges, such as motion artifacts and anatomical variability through technique advancement including flexible biosensors and deep learning-based noise reduction, ultimately enabling personalized cardiovascular monitoring frameworks for scalable clinical translation.

##### Ex vivo diagnostics

Humoral markers from blood, urine, or feces provide valuable clinical insights into vascular aging and CVD progression, thereby constituting essential vascular components required for stage 1 data acquisition. These include inflammatory mediators, plasma proteins (e.g., insulin-like growth factor 1, fibroblast growth factor 21, and fibulin-1), circulating cells (e.g., CD8^+^CD28^−^ T cell subsets), extracellular vesicles, and functional markers like DNA methylation [29,73]. Multi-omics approaches advance biomarker discovery by integrating genomic, transcriptomic, proteomic, and metabolomic data. This comprehensive strategy addresses the limitations of single-dimensional immunoassays and yields a wealth of clinically relevant biomarkers. CVIHEM enables rapid, multiplexed detection critical for precision diagnostics. Functionalized nanomaterials amplify signals through tailored electrical (e.g., field-effect transistors), electrochemical, or optical (e.g., surface plasmon resonance) properties, achieving ultra-sensitive detection with expanded dynamic ranges [57]. Nanotechnology also enables simultaneous detection of multiple cardiac biomarkers and supports the advancement of efficient point-of-care devices [73]. Notably, nanobiosensors are highly capable of detecting novel biomarkers, such as the rapid quantification of specific antigens at single-molecule to nanomolar levels in complex biological fluids [74]. This is crucial as it helps uncover the considerable potential of humoral biomarkers. Beyond prevailing challenges in nanomaterial, unresolved issues persist that require further efforts, particularly in mitigating nonspecific protein adsorption and matrix-induced biofouling in complex biological fluids (Table 1). These challenges necessitate synergistic surface engineering for enhanced molecular discrimination, coupled with AI-powered bioinformatic architectures that systematically correlate multimodal biomarkers with disease pathogenesis, enabling precision cardiovascular diagnostics and prediction across diverse population [57].

#### Stage 2: Integrated analysis and predictive modeling (processing core)

Stage 2 functions as the computational core of CVIHEM, transforming heterogeneous vascular data streams into clinically actionable insights through a system-level pipeline encompassing unified fusion, interpretable modeling, digital twins.

##### Unified fusion

In stage 2, multimodal data including CT angiography (CTA), MRA, PET/CT, ultrasound Doppler, wearable hemodynamics, and ex vivo multi-omics as stated in stage 1 are aligned into a unified representation space to enable coherent analysis across scales. Learned reconstruction and self-supervised denoising elevate signal-to-noise ratio and spatiotemporal resolution under low-dose or low-contrast conditions while reducing acquisition burden. Cross-modal registration and multi-view representation learning establish spatial, temporal, and semantic consistency, allowing imputation for missing modalities with explicit uncertainty propagation [75]. Physical and topological priors are embedded to preserve vascular-tree integrity, hemodynamic constraints, and material parameter ranges so that lesion-vessel relationships remain faithful under pathological variance [76]. A tokenized, multiscale encoding of voxels, waveforms, and molecular features using Transformer and graph-based architectures supports scalable fusion from micro- to macrovascular levels [77]. Despite the above advantages, the approach remains constrained by limited generalization across centers, hallucinated structures due to absent physical constraints, and unstable training (Table 1). Accordingly, future work should prioritize improving generalization and incorporating physics-informed priors to stabilize learning.

##### Interpretable modeling

Interpretable modeling in cardiovascular imaging has evolved from predominantly post hoc approaches to a more integrated paradigm that ties model reasoning to verifiable physiology and clinical entities [78]. Early reliance on saliency maps, SHapley Additive exPlanations values, and concept activation has gradually given way to intrinsically interpretable designs such as concept bottlenecks, prototype-based models, and causal concept learning, which help align latent representations with anatomical regions, lesion phenotypes, and measurable biomarkers [79]. In parallel, counterfactual generation is shifting from visually plausible but physiologically unconstrained edits to formulations that incorporate morphological and hemodynamic feasibility, thereby improving clinical credibility [80]. Alongside interpretability, reliability has become a central focus: Uncertainty calibration methods including temperature scaling, deep ensembles, hierarchical calibration, and selective prediction are increasingly combined with out-of-distribution detection based on maximum SoftMax probability, energy scores, and contrastive feature spaces, although robustness still varies across sites and acquisition protocols [81,82]. Complementing these data-driven advances, physics-based coupling has progressed through computational fluid dynamics (CFD) surrogates, physics-informed neural networks, and graph-based simulators that approximate hemodynamics under controlled assumptions, with multi-fidelity training and synthetic-to-real adaptation accelerating functional index estimation such as fractional flow reserve (FFR) [83,84]. At the signal and tissue mechanics level, waveform analysis that integrates wave intensity methods, forward and reflected wave decomposition, and 1-dimensional to 3-dimensional arterial network models is being linked to wearable photoplethysmography and Doppler measurements to enable individualized parameter inference and cross-setting monitoring [85]. Finally, structured prediction with graph neural networks is maturing toward unified pipelines that use centerline-aware encodings, topology-preserving objectives, and geometric deep learning to maintain vascular connectivity and branching consistency while jointly supporting segmentation, tracking, and anatomical labeling; these advances are increasingly being aligned with reporting systems and governance practices to facilitate prospective validation and workflow deployment. Nevertheless, cross-center stability remains limited, physiologic and morphologic grounding is often weak, and out-of-distribution sensitivity constrains reliability (Table 1). Future work should advance intrinsically interpretable concept models and incorporate multi-modal out-of-distribution detection with cross-modal consistency checks to strengthen grounding and robustness.

##### Digital twins

Digital twin technology spanning lumped-parameter circulation models and reduced-order or graph-based electrophysiology modules is progressing from proof-of-concept pilots to task-focused, patient-specific systems that couple mechanistic simulators with data-driven surrogates [86]. Core capabilities now include multimodal imaging-derived 3-dimensional geometry and centerlines, assimilation of hemodynamic observables such as PWV, central aortic pressure, and Doppler or phase contrast-MRI flow to infer hard to measure parameters such as wall elasticity and microcirculatory resistance, and computation or surrogate estimation of wall shear stress, pressure drops, and functional indices such as FFR [87]. Hybrid mechanistic and statistical modeling spans CFD and fluid structure interaction together with electrophysiology, linked with Bayesian and variational inference, physics-informed neural networks, graph-based simulators, neural operators, and reduced-order models, which enables rapid scenario testing for stent placement, graft sizing, valve or endograft selection, and virtual ablation strategies [88]. Emerging longitudinal modules integrate state space and causal temporal modeling to capture cardiovascular aging, vascular remodeling, and evolution of arrhythmogenic substrates, providing early warning signals and phenotype transitions [89,90]. Despite these advances, cross-modal misalignment, unstable coupling between mechanistic simulators and data-driven surrogates, and unmet real-time constraints still limit clinical reliability (Table 1). Future research should pursue stable joint training with uncertainty awareness and leverage fast-slow surrogates with online assimilation to meet responsiveness and robustness requirements.

#### Stage 3: Translation to clinical and health applications (output layer)

Stage 3 translates processed vascular insights into actionable clinical strategies, prioritizing therapeutic precision, scalability, and proactive health optimization. While stage 2 generates personalized protocols through predictive analytics, their implementation is augmented by engineered medical innovations, such as nanotherapeutics and tissue engineering, that seamlessly bridge analytical findings and clinical action.

##### AI-driven decision strategies

AI-driven decision strategies are central to stage 3 of the CVIHEM framework, operationalizing earlier predictive insights into deployable clinical actions across diagnostics, therapy planning, and proactive health management. In clinical practice, precision diagnostics increasingly couple multimodal imaging with AI-based risk modeling to identify high-risk phenotypes and vulnerable plaques, moving beyond stenosis severity toward biology-informed stratification [91,92]. Recent multicenter studies show that coronary CTA radiomics and deep learning can detect high-risk plaque features and improve short-term major adverse cardiovascular event prediction with external validation across vendors and scanners, supporting integration into chest pain and preventive cardiology workflows [93]. CT-derived functional indices, including FFR and wall shear stress surrogates, demonstrate decision impact in routine settings and correlate with ischemia and outcomes, providing a bridge between anatomical imaging and functional assessment [94]. For therapy planning, cardiovascular digital twins and noninvasive hemodynamics now enable patient-specific scenario simulations for stent sizing and placement, bypass or valve selection, and antiplatelet optimization; hybrid pipelines that combine reduced-order CFD with neural operators have achieved minute-scale screening with calibrated uncertainty, enabling pre-procedural what-if testing and, in early feasibility studies, concordant postoperative hemodynamic improvements [95,96]. In proactive health management, wearable-derived metrics such as central aortic pressure proxies, rhythm burden, and activity signatures are increasingly used to trigger AI-guided lifestyle and pharmacologic interventions; large-scale cohort and real-world studies support scalable arrhythmia burden estimation and blood pressure management with emerging evidence for adherence and outcome benefits [97,98]. Nevertheless, deployment remains limited by complex cross-pathway integration with high workflow burden, challenges in multi-center external validity and bias control, and performance drops after release, motivating future work on model cards with drift monitoring and governance loops, application programming interface (API)-level interoperability, and federated learning with rigorous external validation (Table 1).

##### Novel targets and nanotherapeutics

Inadequate interventions to prevent, delay, or reverse vascular aging is one important reason for the “residual risk” associated with CVDs [99]. Youthful phenotypes can be induced by counteracting vascular aging through mechanisms like enhanced vascular endothelial growth factor signaling, leading to reduced age-related capillary loss, improved organ perfusion and function, and extended lifespan [100]. Eight molecular hallmarks have been identified as key contributors to cardiovascular aging: impaired macroautophagy, loss of proteostasis, genomic instability, epigenetic alterations, mitochondrial dysfunction, cellular senescence, dysregulated neurohormonal signaling, and inflammation [101]. These mechanistically linked hallmarks form an interconnected network, where targeting one affects others, highlighting their therapeutic potential for CVD prevention and treatment [101]. In the CVIHEM framework, nanotherapeutics serve as a promising tool to translate vascular insights developed in stage 2 into enhanced outcomes via precision therapeutics. This can be realized through systemic injection via nanomaterials engineered with targeting ligands, therapeutic cargoes, enzymatic functions, or intelligent designs such as thermo-responsive shape-memory nanobots [9,102]. In addition, heart-related repair can be functionally improved (e.g., mechanically, immunologically, and electrically) with biomaterials incorporating nanomaterials or stem cells and their derivatives [57], whereas vascular tissue engineering has been advanced with biomaterial design and 3-dimensional bioprinting technologies [103]. Data-driven strategies in CVIHEM also facilitate the translation of vascular information into optimized biomaterial parameter designs and vascular architectures, further enhancing translational outcomes. Notably, common challenges in nanomedicine persist as critical barriers in nanotherapeutics (Table 1), underscoring the need for continuous innovation in material design and harmonized clinical translation frameworks to realize translational potential.

##### Nanotheranostics

This approach provides a revolutionary paradigm for CVD management by combining diagnostic and therapeutic functionalities in single nanomaterial platforms. It enables simultaneous real-time monitoring and targeted treatment, markedly enhancing therapeutic efficiency and convenience. Their multifunctionality could be achieved via adaptable nanomaterials loaded with both imageable and therapeutic molecules, or using intrinsic multimodal imaging properties of the nanomaterial itself while enabling therapeutic payload integration and photothermal conversion [57]. For instance, reactive oxygen species-responsive micelles deliver prednisolone for atherosclerosis therapy with lipid-mediated fluorescence tracking [104], while vascular endothelial growth factor receptor 2-targeted nanomaterials provide multimodal (MRI/photoacoustic/ultrasound) imaging-guided anti-angiogenic therapy [105]. Notably, certain nanomaterials, such as IONPs, can serve as imaging agents themselves. When properly engineered, these materials may be developed into multimodal platforms integrating MRI imaging with chemical, physical, or biochemical therapeutic functions [9]. Some examples include bionic nanomaterials co-loaded with therapeutic payloads and γ-Fe₂O₃ nanoparticles for MRI-guided precision therapy [106], and magnetically engineered stem cell constructs achieving myocardial repair with real-time MRI-validated engraftment [107,108]. As an emerging field, nanotheranostics encounters both inherent nanomedicine challenges and unique obstacles such as therapeutic-imaging efficacy mismatch and manufacturing complexity (Table 1). Systematic evaluation of integrated performance metrics and pharmacokinetic/pharmacodynamic relationships will facilitate optimized material selection. Strategic focus should also emphasize clinically viable modular systems with multifunctional precision, balancing innovation pursuit with rational design optimization. Early regulatory engagement and multidisciplinary coordination are critical for translational success [109].

#### Stage 4: Clinical feedback and model refinement (learning loop)

Stage 4 is the learning engine of CVIHEM. It routes real-world data and outcomes from stage 3 back to stage 2 and stage 1, forming a closed loop of “acquire–analyze–translate–feedback”. The paradigm shifts from periodic retraining to online recalibration and federated continual learning, with uncertainty quantification, drift monitoring, and evidence governance, enabling auditable, cross-site upgrades without centralizing raw data.

##### Clinical feedback and optimization for stage 1 (input layer)

To enhance data quality and harmonization across equipment and centers, stage 4 turns quality signals, outcome linked insights, and rescan requests from clinical use into protocol adaptation and calibration actions. For image and waveform quality, learning-based reconstruction and self-supervised denoising are combined with atlas-guided registration to assess signal quality online for whole-body MRA, coronary CTA, ultrasound, and wearable waveforms. The system recommends heart rate gating windows, contrast timing, compressed sensing factors, and reconstruction parameters, and issues prioritized rescans to reduce misses and artifacts [36,37]. For cross-device and cross-site calibration, reconstruction harmonization curves and time synchronization references are established so that imaging timestamps align with physiologic signals (including PWV, photoplethysmography, electrocardiogram, and aortic pressure), reducing topology breaks and functional bias from protocol heterogeneity. In wearable settings, individualized calibration, domain adaptation, and multispectral artifact suppression help mitigate the influence of skin tone, body habitus, and motion on blood pressure and rhythm estimation; multi-center real world assessments strengthen generalizability [72,110]. For nano and in vitro diagnostics, peripheral inflammatory and immune phenotypes (e.g., extracellular vesicles, proteomics, and metabolomics) are cross-validated against imaging signatures of plaque vulnerability and against MPI or ferumoxytol-enhanced MRA to refine probe dosing, timing, and readout windows. Sensor drift and biofouling in nano-biosensors are corrected via surface modification and reference channels, alongside prospective safety follow-up and registry style evidence building to support translation [111,112]. Together, this “quality–protocol–calibration–verification” loop reinforces stage 1 stability and reproducibility across devices, centers, and home environments.

##### Clinical feedback and optimization for stage 2 (processing core)

Stage 2 converts measurable acquisition side noise and protocol differences, as well as application side strategy and outcome signals, into actionable triggers for retraining, recalibration, and data assimilation. This closes gaps that historically led to vascular topology breaks, hallucinated imputations, threshold misalignment, and site-specific drift. In unified fusion and cross-modal alignment, protocol metadata from stage 1 (such as signal-to-noise ratio/contrast-to-noise ratio, gating windows, injection to acquisition timing, and reconstruction versions) are used to reweight registration and fusion losses and to perform spatiotemporal resampling, thereby limiting the impact of low contrast and motion segments on vascular continuity. In multi-center settings, site-specific adapters and style harmonization reduce semantic drift, with independent validation on public datasets to stabilize geometric and semantic consistency. For missing modalities, the system first produces uncertainty-bounded interval predictions, then backfills and retrains when supplementary data arrive, reducing persistent hallucinations under distribution shift [113]. In interpretability and reliability, stage 3 strategies and outcomes (procedures, devices, medication intensity, etc.) map to concept bottlenecks and prototype structures aligned with clinical entities, building a traceable evidence chain, and minimizing visually plausible but physiologically inconsistent matches [114,115]. Counterfactuals generate physiologically plausible alternatives to update decision boundaries and thresholds based on follow-up differences. Selective prediction with conformal prediction provides coverage-controlled pathways for release, escalation, human review, or safe fallback in high-uncertainty regions [116]. In digital twins and physics-based assimilation, observations (PWV, aortic pressure, Doppler flows, FFR, and 4-dimensional flow wall shear stress) feed Bayesian or variational schemes to update wall elasticity, microcirculatory resistance, and boundary conditions online. For robustness and federated continual learning, multi-center collaboration uses personalized federated learning with differential privacy and secure aggregation to mitigate privacy risks and site heterogeneity. Collectively, this “fusion–interpretability–assimilation–federation” loop yields outcome-aware, strategy-aligned adaptive optimization of stage 2 in real-world practice.

##### Clinical feedback and optimization for stage 3 (clinical and health applications)

Stage 3 focuses on deeply embedding model outputs into clinical workflows while writing back strategy and outcome evidence to sustain the loop. To meet bedside, intraprocedural, and wearable constraints, models are distilled, pruned, and quantized for low-latency inference [117]. Uncertainty-gated execution triggers release, escalation, human review, or safe fallback, shortening the information loop while maintaining safety. Sequential modeling of the care pathway quantifies deviations between AI recommendations and actual choices, device or drug adjustments, readmissions, and complications, producing estimates of net benefit and potential harm that drive online threshold and rule updates [118]. For individualized therapy, hybrid mechanistic–statistical updates connect postoperative hemodynamic metrics (such as wall shear stress, pressure drops, and coronary flow reserve) and mid-term outcomes (e.g., 6-month patency and perfusion improvement) to the strategy optimizer, which in turn steers digital twins and follow-up scheduling. On-device measures including proxies of central aortic pressure, rhythm burden, and activity signatures further support event-driven follow-up and closed-loop lifestyle and pharmacologic interventions, with real-world evidence suggesting favorable adherence and effectiveness [119].

### The uniqueness of CVIHEM

As mentioned earlier, various CVIHEM techniques have been developed for vascular assessment or treatment. Nevertheless, most of them are frequently centered on a single point, either capturing certain vascular information or targeting specific abnormalities, resulting in limited clinical benefits. CVIHEM distinguishes itself by synergizing multimodal technologies (Table 2) through a vascular network-centric framework that integrates and translates high-dimensional spatiotemporal data. Whereas precision medicine emphasizes genomics, medical imaging focuses on anatomy, wearables track basic vitals, and digital twins rely on simulated models, CVIHEM uniquely converges these domains via engineering–medical integration to enable continuous vascular dynamics modeling, predictive therapy optimization, and personalized health management. This multimodal fusion and iterative learning architecture bridge isolated data paradigms, offering end-to-end translational capabilities that unify diagnosis, intervention planning, and proactive care within a single adaptive system.

**Table 2.** The uniqueness of CVIHEM in comparison to other relevant techniques

Technique/field	Scope	Data integration capability	Clinical translation focus	Limitations addressed by CVIHEM
Precision medicine	Genomics-driven therapeutics	Low-to-moderate (static genomics)	Drug targeting	Siloed data; lacks real-time vascular dynamics
Medical imaging (e.g., MRI/CT)	Anatomical visualization	Moderate (isolated snapshots)	Diagnosis	Noncontinuous; insufficient functional insight
Cardiovascular digital twin	Organ/system-scale simulation	High (virtual replicas)	Predictive modeling	Limited by input data granularity
Current wearables	Vital sign monitoring	Low (e.g., heart rate/activity)	Basic wellness tracking	Superficial metrics; insufficient vascular specificity
CVIHEM	Vascular and related systems-centric	High (spatiotemporal + multimodal)	Diagnosis + therapy + health management	Bridges gaps through collaborative integration of engineering, medicine, and allied disciplines

CT, computed tomography; CVIHEM, cardiovascular information and health engineering medicine; MRI, magnetic resonance imaging

### Technological development pathway of CVIHEM

The evolution of CVIHEM follows a structured trajectory to systematically address technical barriers (Table 1) while advancing its core mission toward a closed-loop vascular health ecosystem.

In the short term, efforts will prioritize addressing core limitations in vascular data acquisition. Ferumoxytol-enhanced angiography requires algorithmic advances to resolve subvoxel vessel detection and arteriovenous synchronization challenges. Multimodal imaging integration (e.g., MRI–CT–ultrasound fusion) will prioritize standardized datasets and generative adversarial networks to harmonize modality heterogeneity. In addition, wearable monitoring systems demand flexible biosensor materials and deep learning-based noise reduction to mitigate motion artifacts and calibration frequency. For nanomaterial-based diagnostics, surface functionalization strategies are needed to enhance biomarker sensitivity in complex biological matrices. These efforts collectively aim to establish a robust input layer for downstream analysis. Concurrently, near-term efforts will also prioritize multimodal foundation models pretrained on CTA/MRA/ultrasound/physiologic data, with knowledge distillation for edge deployment in wearable devices, ensuring clinical adaptability while maintaining predictive accuracy. Early validation of explainable AI frameworks for vascular risk prediction will complement these efforts, countering algorithmic opacity through physiology-aware constraint integration.

Mid-term developments will focus on integrative modeling and translation. Mid-term progress hinges on resolving model robustness and clinical applicability. Unified fusion frameworks (stage 2) require physics-informed priors to prevent hallucinated vascular structures, while interpretable modeling will integrate multi-modal out-of-distribution checks to align with physiological constraints. Digital twin development (stage 3) must overcome real-time latency through fast-slow surrogate modeling and online data assimilation techniques. In parallel, nanotherapeutics optimization will balance sustained release profiles with scalable synthesis, supported by pharmacokinetic/pharmacodynamic systematic studies. Cross-disciplinary consortia will validate AI models across heterogeneous populations to reduce deployment instability, addressing root causes like data heterogeneity and pathological overfitting.

Long-term goals aim to establish a closed-loop vascular health ecosystem, anchored by self-optimizing digital twins with lifelong learning capabilities. Long-term success demands systemic innovation. A federated learning architecture (stage 4) will resolve cross-institutional interoperability through modular APIs and AI-driven compliance monitoring, enabling continuous model refinement via real-world feedback. Nanomaterial clinical translation requires stealth coatings and heavy metal-free designs to mitigate toxicity risks, validated through early regulatory engagement. The CVIHEM paradigm itself will evolve into a standardized closed-loop system, integrating engineering–medical collaboration platforms and validation protocols. Ultimately, these efforts aim to realize precision vascular health management, where multidimensional data acquisition, analysis, and feedback converge into a unified, prevention-oriented framework.

## Conclusion

In conclusion, CVIHEM offers a practical solution for the accurate acquisition, integration, processing, and efficient utilization of vascular information. By leveraging this innovative framework and its advanced technologies, CVIHEM aims to develop novel materials, formulations, devices, and intelligent systems tailored for clinical applications. With its substantial potential to enhance preventive, diagnostic, therapeutic, and rehabilitative strategies, CVIHEM is committed to expanding the capabilities of clinical medicine and advancing the field of vascular healthcare.
