# Supplementary material for: Cardiovascular Information and Health Engineering Medicine
Source: Research (Wash D C). 2025 Oct 17;8:0956. doi: 10.34133/research.0956 (PMC12531494; doi:10.34133/research.0956)
Supplement: Supplementary 1 — Table S1 [file research.0956.f1.docx]

**Supplementary Table 1. Recent advances of multimodal fusion and Al techniques for CVDs and related medical applications.**

| **Method** | **Modalities** | **AI techniques** | **Applications** |
| --- | --- | --- | --- |
| Franke et al. [47] | MRI/MPI | co-registration, spatio-temporal analysis | blood flow quantification |
| Sui et al. [48] | rs-fMRI/sMRI/dMRI | multimodal networks | neuromarkers |
| Wang et al. [53] | SAX/4CH Cine/LGE MRI | video-based swin transformer | screening and diagnosis CVDs |
| Zhou et al. [54] | Radiograph/ chief complaint | transformer, bidirectional attention | multimodal fusion |
| Yao et al. [50] | MRA/CTA | graph network | vessel labeling |
| Guo et al. [55] | TOF MRI/MRA/CTA | 2D-3D fusion, weakly-supervised learning | vessel segmentation |
| Xue et al. [56] | T1w/T2w/DWI/SWI/FLAIR | transformer, multimodal data embeddings | vascular dementia diagnosis |

2D-3D, two-dimensional to three-dimensional; 4CH, 4-chamber; AI, artificial intelligence; CVDs, cardiovascular diseases; CT, computed tomography; CTA, computed tomography angiography; DWI, diffusion-weighted imaging; dMRI, diffusion MRI; FLAIR, fluid-attenuated inversion recovery; fMRI, functional MRI; LGE, late gadolinium enhancement; MRA, magnetic resonance angiography; MRI, magnetic resonance imaging; MPI, magnetic particle imaging; rs-fMRI, resting-state functional MRI; SAX, short-axis; sMRI, structural MRI; SWI, susceptibility-weighted imaging; T1w, T1-weighted; T2w, T2-weighted; TOF, time-of-flight.
